# Weil's Disease

**Published:** 1890-11-15

**Authors:** 


### WEIL'S DISEASE.

This name has been given to a disease of somewhat frequent occurrence in Egypt, Syria, and the south-east of Europe, hitherto vaguely designated as typhomalarial, typhus icterodes, bilious typhus (Griesinger), and icterodes kartulis. By some it is considered to be a form of relapsing fever, intensified by previous malarial influences; but there is a growing tendency to recognise it as a distinct, specific, and infectious disease, probably produced by the use of polluted marsh waters. The Viennese, Hungarian, and Slavonian medical journals have recently contained accurate reports of a number of cases, the remarkable uniformity of the symptoms and *post-mortem* appearances in which seem to entitle it to a distinct place in nosology, although doubtless akin to relapsing fever. It sets in suddenly with rigors, fever, headache, and vomiting. Jaundice appears between the third and fifth day, followed by marked enlargement of the liver, spleen, and inguinal glands. There are pains in the limbs, fœtor of the breath, albuminuria, and hæmaturia, and in severe cases bloody stools. Death usually occurs on the tenth day, and in the mildest cases recovery is extremely slow. The *post mortem* appearances are general icterus, fatty degeneration of the heart, hæmorrhagic pachymeningitis, the liver and spleen enlarged, and the former stained with bile, acute hæmorrhagic interstitial nephritis, and internal hæmorrhages in various organs. Dr. Frantisek Sumbera reports three cases, all fatal, occurring in tradesmen aged between 40 and 50 years. Staff-Surgeon M. Hueber, four of Austrian soldiers aged 22 to 26, all previously healthy men, one only fatal. In these angina was an early and distressing symptom. Dr. Sezary, of Algiers, one of a butcher aged 45, who made a tedious recovery; and Dr. Justyn Karlinski, of Stolac, in Herzegovina, five cases of persons who had previously suffered severely from malarial fevers. One died, but the others recovered fairly soon. Relapsing fever was prevalent in the district, and the brother of one patient was in the hospital with it at the time. He is inclined to doubt its specific character, believing it under these circumstances to be but an intensified form of relapsing fever occurring in persons whose constitutions have been broken down by malarial fever. He found in the blood of all the organs spirilli very similar to those of relapsing fever, but shorter and more comma-shaped. Sumbera and Hueber failed to detect any such organisms.

It is possible that Karlinski has rightly interpreted the nature of his cases which from their speedy recovery, although in previous weak health, seem to have been different from those of Sumbera and Hueber. And Dr. Ducamp, of Montpellier, has described an illness with somewhat similar symptoms which attacked six men who had been engaged in clearing an obstructed sewer. One of these died, but it was probably only a form of septic poisoning.

